# Detection of liver mitochondrial oxaloacetate by NMR spectroscopy and membrane potential‐dependent accumulation

**DOI:** 10.1096/fj.202500039R

**Published:** 2025-04-01

**Authors:** Liping Yu, Brian D. Fink, William I. Sivitz

**Affiliations:** ^1^ Department of Molecular Physiology and Biophysics University of Iowa and the Iowa City Veterans Affairs Medical Center Iowa City Iowa USA; ^2^ Department of Biochemistry and Molecular Biology University of Iowa and the Iowa City Veterans Affairs Medical Center Iowa City Iowa USA; ^3^ NMR Core Facility University of Iowa and the Iowa City Veterans Affairs Medical Center Iowa City Iowa USA; ^4^ Department of Internal Medicine/Endocrinology and Metabolism University of Iowa and the Iowa City Veterans Affairs Medical Center Iowa City Iowa USA

**Keywords:** inner membrane potential, liver, metabolites, mitochondria, oxaloacetate, respiration

## Abstract

Oxaloacetate (OAA) is a central liver metabolite fundamental to critical metabolic pathways. However, understanding OAA metabolism in the liver has been limited because the compound is very difficult to measure by mass spectroscopy and not abundant enough for detection by other methods. Here we describe a novel approach to quantifying OAA in liver mitochondria. Moreover, we provide evidence for membrane potential‐dependent OAA accumulation in mitochondria during complex II—energized respiration consistent with OAA inhibition of succinate dehydrogenase.

## Graphical abstract

Oxaloacetate is centrally positioned in the TCA cycle to regulate critical steps towards whole‐body physiology. Nonetheless, studies of OAA metabolism in the liver are severely limited because OAA is unstable and very difficult to measure by mass spectrometry and too low to detect by conventional procedures.

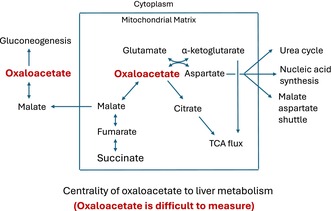

## 1 INTRODUCTION

Oxaloacetate (OAA) is critically positioned in the TCA cycle to react with acetyl‐CoA to form citrate or with glutamate to generate aspartate and α‐ketoglutarate or to act as a reactant in the malate dehydrogenase reaction affecting malate exit from mitochondria and regeneration of OAA for gluconeogenesis. The importance is clear since hepatic TCA flux is basic to fat and glucose metabolism; because aspartate is critical to nucleic acid synthesis, the malate–aspartate shuttle, and the urea cycle; and because α‐KG leads to downstream TCA flow to succinyl‐CoA.

Nonetheless, studies of OAA metabolism in the liver have been severely limited because OAA is unstable^1^ and very difficult to measure by mass spectroscopy and too low to detect by conventional procedures. Although low in abundance, products of its metabolism (citrate, aspartate, α‐ketoglutarate, malate) are far more abundant, implying that OAA turnover is critical to cellular metabolism. Moreover, OAA is a well‐known, highly potent inhibitor of succinate dehydrogenase (SDH).^2^, ^3^

We previously reported that in mitochondria of muscle or brown adipose tissue (BAT) respiring on the complex II substrate, succinate, the accumulation of OAA is dependent on inner membrane potential (ΔΨ), as we showed by manipulating ΔΨ with incremental clamped concentrations of ADP or by chemical uncoupling.^4^, ^5^, ^6^ When succinate‐energized muscle mitochondria were titrated in this way, respiration initially increased as ΔΨ decreased. However, at a certain point, although ΔΨ continued to drop, OAA began to accumulate associated with decreased respiration, likely from OAA inhibition of SDH.^4^, ^5^ However, with the same methodology, we were unable to detect OAA in liver, a disappointing scenario given the centrality of OAA to liver metabolism.

Here we describe a novel methodology to resolve this issue, enabling measurement of liver mitochondrial OAA production. We further report the consequences of OAA production on complex II energized respiration.

## 2 METHODS

### 2.1 Detection of liver mitochondrial OAA

Typically, studies of mitochondrial respiration are done using an oxygen electrode in a closed‐toto‐air chamber, e.g., the Oxygraph‐2k respirometer which we used herein. Mitochondria are generally incubated in amounts of about 175 μg mitochondrial protein/mL. In our hands, incubating muscle or BAT (but not liver) mitochondria in this way enables robust assessment of OAA production from labeled succinate depending on ΔΨ. To try to detect OAA in liver mitochondria, we increased the number of organelles incubated. However, that caused very rapid depletion of oxygen within the medium without enough time to detect metabolites.

Therefore, we used the Oxygraph with the chamber open to air and large numbers of mitochondria to assess OAA production by 2‐dimensional NMR spectroscopy. We incubated 1050 μg mitochondria/mL (6‐fold more than usual) energized by succinate at different levels of clamped [ADP]. In this way, we could incubate mitochondria for 10 min, avoiding loss of O_2_ tension to the point of impaired mitochondrial O_2_ consumption, which is known to be robust down to 20% of baseline (atmospheric equilibration).

### 2.2 Mitochondrial respiratory rate

The Oxygraph output for oxygen consumption rate (OCR) assumes a closed respiratory chamber.^7^ If open, this will be inaccurate since the OCR is calculated from the derivative of the oxygen content, which drops over time. However, the electrode does continuously and accurately record the O_2_ concentration. As mitochondria consume oxygen in an open chamber, the media concentration represents the balance between that absorbed and that replaced by the atmosphere. Hence, the OCR over any small time interval is the mean difference between measured and baseline over the time in question (approximate integration). Because there may be some lag time, we take this value as “estimated” OCR. The Oxygraph measures oxygen concentrations every 2 s; hence, we calculated estimated OCR per second by dividing by 2. For calculations, we exported the Oxygraph data to Microsoft Excel. Even if we do not know the exact lag time, the data comparing one condition to another should be affected in like fashion.

### 2.3 Mitochondrial inner membrane potential

ΔΨ cannot be measured in an open chamber. To show the relation of respiration to ΔΨ in liver mitochondria, we incubated 100 μg/2 mL in a closed chamber with a tetraphenylphosphonium electrode, as we have in the past.^4^, ^8^, ^9^

### 2.4 Isolation and purification of mitochondria

Mitochondria were prepared by differential centrifugation and purified using a Percoll gradient as we described.^10^

### 2.5 Incubation of mitochondria

Mitochondria were incubated at 37°C in 2 mL of ionic respiratory buffer (105 mM KCl, 10 mM NaCl, 5 mM Na_2_HPO_4_, 2 mM MgCl_2_, 10 mM HEPES pH 7.2, 1 mM EGTA, 0.2% defatted BSA).

### 2.6 ADP energy clamp

This was done by a method we previously described.^10^, ^11^ Hexokinase and 2‐deoxyglucose (2DOG) are added to respiratory media, causing rapid and irreversible recycling of ATP back to ADP as 2DOG consumes ATP to generate 2DOG phosphate. ADP‐dependent ΔΨ is therefore also held constant.

### 2.7 Metabolite measurements

Metabolites including oxaloacetate were determined in Oxygraph chamber contents by 2‐dimensional NMR as we previously described.^4^, ^5^

## 3 RESULTS AND DISCUSSION

**FIGURE 1:**
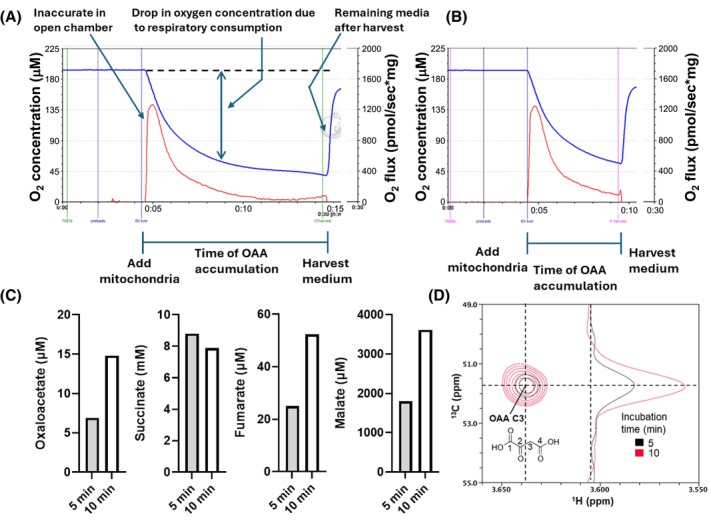
Mitochondrial O_2_ flux and metabolite accumulation for complex II energized respiration in an open to air chamber. (A) Computer printout of Oxygraph data wherein we incubated 1050 μg/mL of liver mitochondria (6 × usual amounts per conventional run) energized by 10 mM succinate in an open respiratory chamber at a clamped [ADP] of 512 μM. Mitochondria were added as shown and incubated for 10 min before harvesting the chamber contents for assessment of metabolites by NMR spectroscopy. Blue curves depict O_2_ concentration versus time. Red depicts O_2_ flux as assessed by the Oxygraph programing. However, in this experiment, the red curve does not correctly represent O_2_ flux because the chamber is open to air. The actual O_2_ consumption is depicted as the double arrow representing the drop in O_2_ concentration at the time point shown. (B) Data as in panel A but over a 5 min incubation time. (C) Metabolite measurements by NMR at harvest in panels A and B including oxaloacetate. (D) Overlayed contour plot of the OAA C3 cross‐peak region of the ^13^C/^1^H HSQC NMR spectra of the harvested media used to measure oxaloacetate at 5 and 10 min as shown in panel C. Vertical 1D slices through the OAA C3 cross‐peaks are also shown for display of the cross‐peak intensities.

Figure 1A,B depict tracings for liver mitochondria (1050 μg/mL) incubated in an open chamber for 5 or 10 min before harvesting media for metabolite determinations. 512 μM ADP was used based on our past work in muscle and BAT showing that OAA accumulation is ΔΨ‐dependent, most evident at lower levels, i.e., higher ADP or chemical uncoupling.^4^, ^8^, ^9^ Figure 1C shows the metabolite data for harvested samples of panels A and B. Figure 1D shows overlays of the 2‐dimensional (2D) ^13^C/^1^H HSQC NMR spectra of the OAA C3 region for quantification of OAA. The data reveal that OAA is clearly detectable and accumulates over time, as do the precursors fumarate and malate, while succinate decreases as it is the energizing substrate.

**FIGURE 2:**
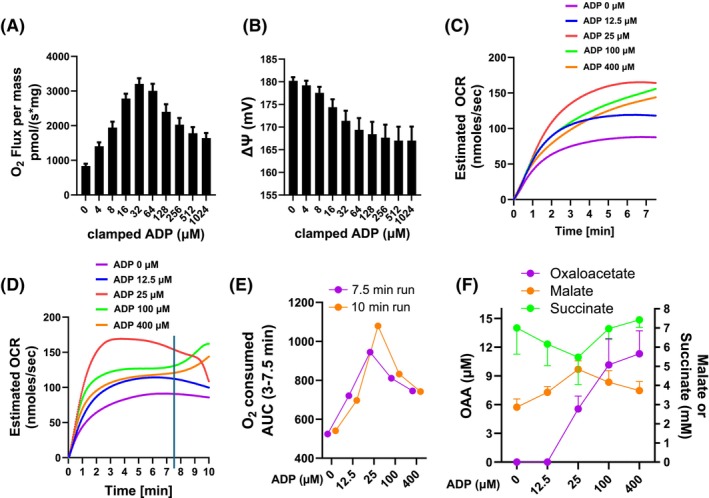
ΔΨ‐dependent respiration and OAA accumulation. (A and B) Respiration and ΔΨ by liver mitochondria, 100 μg/2 mL, in a 2 mL closed Oxygraph chamber volume energized with 10 mM succinate at different levels of ADP, *n* = 3, data show mean ± SE. (C) Estimated oxygen consumption rates by liver mitochondria, 1050 μg/mL, in a 2 mL open Oxygraph chamber volume energized with 10 mM succinate for 7.5 min at different levels of ADP before harvesting media for metabolite measurements. (D) Respiration as in panel C except that respiration was measured for 10 min. The data beyond 7.5 min for ADP of 25 μM or more is difficult to understand. If O_2_ tension drops enough respiration will fail allowing atmospheric air to raise O_2_ tension resulting in an apparent artifactual rise in calculated respiration. This likely occurred at 25 μM ADP. At 100 and 400 μM ADP respiration appears to rise after 7.5 min for unclear reasons, possibly a change in OAA (high at this time) and SDH dynamics. (E) Oxygen consumed (area under the curve, AUC) from 0 to 7.5 min by the mitochondria of panels C (7.5 min, purple) and D (10 min, orange). (F) Metabolites determined after respiration in panels C and D (mean ± SD).

Figure 2A,B show the relationship of respiration and ΔΨ in liver mitochondria (100 μg/2 mL) incubated in a closed chamber. The data demonstrate the biphasic response of complex II‐energized respiration, as we previously reported for skeletal muscle and BAT mitochondria.^4^, ^6^ Figure 2C,D show the curves for two experiments depicting estimated OCR rate versus time (1050 μg mitochondria/mL in an open chamber) for 7.5 or 10 min at different levels of clamped ADP. Figure 2E shows the total oxygen consumed in the first 7.5 min for the two experiments. Figure 2F shows the metabolite levels measured at the end of the Oxygraph runs at different levels of clamped ADP. Some of the variation in Figure 2F is due to the harvesting at different times, i.e., 7.5 or 10 min, respectively. The data beyond 7.5 min in Figure 2D are difficult to interpret (see figure legend).

Taken together, the data in Figure 2 demonstrate that respiration energized at complex II is dependent on ΔΨ in a biphasic pattern, with OAA accumulation increasing with decreasing ΔΨ (i.e. higher ADP concentrations). OAA is a very potent inhibitor of SDH at very low molar amounts, as we and others have shown.^3^, ^4^ Hence, the data are consistent with and suggestive that OAA inhibition of SDH, at least in part, explains the biphasic ADP‐dependent pattern. Although not addressed here, our past work^4^ showed evidence that OAA accumulation at lower ΔΨ is due to less ROS generation (known to be ΔΨ‐dependent). ROS maintains NADH in the reduced state^12^ driving the malate dehydrogenase reaction to the left. Less ROS enables rightward flow and OAA production.

A limitation is that ΔΨ cannot be determined simultaneously with respiration. Also note that OAA can be measured by commercially available kits instead of our NMR method, although sensitivity is no better than our NMR technique at about 1–2 μM. Moreover, kit methods should be less specific, especially at the concentrations observed herein, which would be at the very low linear ends of the curves used in kits. Further, by NMR we can assess multiple metabolites beyond OAA in the same sample. Access to NMR is required but is available at most centers where TCA metabolites are assessed. In our hands, the time, complexity, and cost are no greater and much less if multiple metabolites are quantified.

In summary, we demonstrate a novel method for detecting OAA production by liver mitochondria and provide evidence for OAA inhibition of complex II. Although we have shown similar past findings for muscle and IBAT mitochondria, we believe the liver is of special interest due to its centrality to whole body physiology as discussed above. Our methodology can now be applied to more extensive studies on hepatic mitochondrial function, e.g., as related to gluconeogenesis, fat metabolism, and neoplastic disease as well as in studies of animal disease‐state models.

## AUTHOR CONTRIBUTIONS

L. Yu, B. D. Fink, and W. I. Sivitz conceived and designed the research; L. Yu and B. D. Fink performed the research; L. Yu, B. D. Fink, and W. I. Sivitz analyzed the data; L. Yu and W. I. Sivitz wrote the paper.

## FUNDING INFORMATION

NIH NIDDK 1 R01 DK123043‐01A1, William Sivitz.

## DISCLOSURES

The authors have stated explicitly that there are no conflicts of interest in connection with this article.

5

## Data Availability

Data will be shared upon request by contacting William Sivitz, william-sivitz@uiowa.edu, University of Iowa, Division of Endocrinology and Metabolism, Iowa City IA, 52246, USA.
